# Gag-Specific CD4 and CD8 T-Cell Proliferation in Adolescents and Young Adults with Perinatally Acquired HIV-1 Infection Is Associated with Ethnicity — The ANRS-EP38-IMMIP Study

**DOI:** 10.1371/journal.pone.0144706

**Published:** 2015-12-09

**Authors:** Jérôme Le Chenadec, Daniel Scott-Algara, Stéphane Blanche, Céline Didier, Thomas Montange, Jean-Paul Viard, Catherine Dollfus, Véronique Avettand-Fenoel, Christine Rouzioux, Josiane Warszawski, Florence Buseyne

**Affiliations:** 1 CESP INSERM U1018, Le Kremlin-Bicêtre, France; 2 Institut Pasteur, Unité de Régulation des Infections Rétrovirales, Paris, France; 3 AP-HP, Unité Immunologie et Hématologie Pédiatrique, Hôpital Necker-Enfants Malades, Paris, France; 4 Institut Pasteur, Unité d’Epidémiologie et Physiopathologie des Virus Oncogènes, Paris, France; 5 CNRS, UMR 3569, Paris, France; 6 EA7327, Université Paris Descartes, Sorbonne Paris Cité, Faculté de Médecine, Paris, France; 7 AP-HP, Centre de Diagnostic et de Thérapeutique, Hôpital de l’Hôtel-Dieu, Paris, France; 8 AP-HP, Service d’Hématologie et d’Oncologie Pédiatrique, Hôpital Trousseau, Paris, France; 9 AP-HP, Laboratoire de Virologie, Hôpital Necker-Enfants Malades, Paris, France; 10 Université Paris-Sud, Le Kremlin-Bicêtre, France; University of Hawaii, UNITED STATES

## Abstract

The ANRS-EP38-IMMIP study aimed to provide a detailed assessment of the immune status of perinatally infected youths living in France. We studied Gag-specific CD4 and CD8 T-cell proliferation and the association between the proliferation of these cells, demographic factors and HIV disease history. We included 93 youths aged between 15 and 24 years who had been perinatally infected with HIV. Sixty-nine had undergone valid CFSE-based T-cell proliferation assays. Gag-specific proliferation of CD4 and CD8 T cells was detected in 12 (16%) and 30 (38%) patients, respectively. The Gag-specific proliferation of CD4 and CD8 T cells was more frequently observed in black patients than in patients from other ethnic groups (CD4: 32% vs. 4%, *P* = 0.001; CD8: 55% vs. 26%, *P* = 0.02). Among aviremic patients, the duration of viral suppression was shorter in CD8 responders than in CD8 nonresponders (medians: 54 vs. 20 months, *P* = 0.04). Among viremic patients, CD8 responders had significantly lower plasma HIV RNA levels than CD8 nonresponders (2.7 vs. 3.7 log_10_ HIV-RNA copies/ml, *P* = 0.02). In multivariate analyses including sex and HIV-1 subtype as covariables, Gag-specific CD4 T-cell proliferation was associated only with ethnicity, whereas Gag-specific CD8 T-cell proliferation was associated with both ethnicity and the duration of viral suppression. Both CD4 and CD8 responders reached their nadir CD4 T-cell percentages at younger ages than their nonresponder counterparts (6 vs. 8 years, *P* = 0.04 for both CD4 and CD8 T-cell proliferation). However, these associations were not significant in multivariate analysis. In conclusion, after at least 15 years of HIV infection, Gag-specific T-cell proliferation was found to be more frequent in black youths than in patients of other ethnic groups, despite all the patients being born in the same country, with similar access to care.

## Introduction

The children infected with HIV at the beginning of the epidemic are now reaching adolescence and adulthood [1]. Despite the tremendous clinical benefits of combined therapy, suboptimal immune restoration may account for the high rates of some cancers or weaker responses to vaccines in these individuals [2, 3]. Immune restoration in infants and children is governed by specific features of HIV pathogenesis, such as viral replication and thymic activity, both of which are higher in these patients, and by treatment issues specific to pediatric patients, such as the earlier initiation of ART to prevent rapid clinical progression, and poorer adherence [4–6]. Immune restoration has been poorly characterized, both qualitatively and quantitatively, in young adults who were infected during the perinatal period.

Even in successfully treated patients, HIV-specific CD4 and CD8 T lymphocytes exert some control over replication levels [7–12]. In future therapeutic strategies targeting the viral reservoir, HIV-specific T cells may play an important role in the destruction of infected cells after the reversal of viral latency [13, 14]. A knowledge of the frequency and function of these cells in patients treated for more than a decade is required. The restoration of Gag-specific T cells may differ between treated children and adults, because thymopoiesis is more vigorous in younger patients [4, 15, 16]. In treated children, antiretroviral therapy induces a diversification of the CD8 T-cell repertoire that is positively correlated with the restoration of T-cell proliferation [17].

The ANRS-EP38-IMMIP study aimed to provide a detailed assessment of the immune status of perinatally infected youths living in France. We previously reported that the levels of naive CD4 T cells and recent thymic emigrants in these individuals were within the range reported for uninfected youths [18]. We present here our findings for Gag-specific CD4 and CD8 T-cell proliferation, two immune correlates of viral control in HIV-infected adults [19, 20]. The HIV disease history of these patients was known since their birth or initial care in infancy, making it possible to determine whether the association between Gag-specific T-cell proliferation and HIV disease was consistent with specific hypotheses concerning HIV-specific T-cell restoration. The three specific hypotheses tested were: (1) The initiation of effective therapy at a younger age enhances the restoration of HIV-specific T cells, as younger patients have stronger thymic activity and a shorter duration of exposure to the destructive effects of viral replication; (2) More severe or longer term immunodeficiency and greater disease severity impair the restoration of HIV-specific T cells, as some immune damages are irreversible or only partially reversed by the suppression of viral replication; (3) The association between Gag-specific T-cell proliferation and viral levels differs between patients with suppressed and active viral replication. In aviremic patients, who have no antigenic stimulation at the time of testing, we would expect Gag-specific T-cell proliferation to be stronger in patients who have experienced recent episodes of viral replication. In viremic patients, in whom the antigen is present, we would expect Gag-specific T-cell proliferation to be inversely correlated with the level of HIV replication.

## Materials and Methods

### Ethic statement

This study was approved by the “Comité de protection des personnes Ile-de-France II” (registration number 06-09-08), authorized by the “Direction Générale de la Santé” (authorization number 2006-AOO142-49), and registered as an observational study at www.clinicaltrials.gov under identifier NCT01055873. All patients, and their legal guardians for those under 18 years of age, received written information and signed an informed consent form.

### T-cell proliferation assay

Peripheral blood mononuclear cells (PBMCs) were isolated from heparin-treated blood by density centrifugation, and were immediately labeled by incubation with 10 μM CFSE (5-6-carboxyfluorescein diacetate succimidyl ester, Invitrogen, Life Technologies Corporation, Saint-Aubin, France) in serum-free medium for 30 minutes at 37°C. They were seeded in Dulbecco’s minimal essential medium-4500 mg/l Glucose-GlutaMAX I (Invitrogen) supplemented with 10% human serum (Biowest, Abcys, Paris, France), at a density of 10^6^ cells/ml, and stimulated with a pool of Gag peptides (15-mer peptides covering the clade B consensus sequence, NIH #8117). The peptide pool, containing 122 peptides, each at a concentration of 0.4 μg/ml, was found to be optimal for PBMC stimulation in titration experiments (data not shown). Peptide diluent (0.034% DMSO, Pierce, Thermo Fisher Scientific, Villebon-sur-Yvette, France) was used as a negative control, and the enterotoxin B of *Staphylococcus aureus* (SEB, 500 ng/ml, Sigma-Aldrich, Saint-Quentin Fallavier, France) was used as a positive control. For a subset of 17 patients, PBMCs were stimulated with the p24^gag^ protein and the control protein at a concentration of 5 μg/ml (Protein Sciences Corporation, Cat nos. 2004 and 1500), and with the peptide pool and diluent, for comparison of the two antigenic stimuli. Six days later, PBMCs were labeled with a combination of anti-CD3-ECD, anti-CD4-PC7, and anti-CD8β-PC5 antibodies (Beckman Coulter, Villepinte, France). Cells were analyzed on a FC500 flow cytometer (Beckman Coulter). The CD4 and CD8 T-cell subpopulations were defined as CD3^+^CD4^+^ and CD3^+^CD8β^+^ cells, respectively. Unstimulated cells were used to position the CFSE^low^ gate. Then, for each set of antigen-stimulated cells, CFSE^low^ T cells were quantified among CD4 and CD8 T cells. The difference in the percentages of CFSE^low^ cells between antigen-stimulated and mock-stimulated cells and the ratio of these percentages (stimulation index, SI) were calculated. PBMCs from eight uninfected donors were tested with HIV Gag antigens. The mean + 2SD antigen-specific T-cell proliferation for these control subjects was 0.8% for the difference and 4 for the ratio. These values were used as a dual cutoff criterion, to define a positive response in the T-cell proliferation assay. Patients with positive results in the Gag-specific CD4 T-cell assay are referred to as CD4 responders (CD4Rs) and those with negative results for this assay are referred to as CD4 nonresponders (CD4NRs). Similarly, CD8Rs and CD8NRs are defined on the basis of the results of the Gag-specific CD8 T-cell proliferation assay.

### T-lymphocyte phenotyping

CD4 and CD8 T-lymphocyte phenotypes were determined on fresh whole blood, using combinations of the following antibodies: CD28-FITC, CD4-RD1, CD45RA-ECD, CD45RO-ECD, CD62L-PC5, CD27-PC5, CD4-PC7, and CD8-PC7 (Beckman Coulter, Villepinte, France), as described in [18]. CD4_N_ levels were defined as the percentage of CD4 T lymphocytes that were CD45RA^+^CD62L^+^, CD8_N_ levels as the percentage of CD8 T lymphocytes that were CD45RO^-^CD28^+^CD27^+^. For 57 patients, frozen PBMCs were available for the quantification of CD4_RTE_, with Live-Dead-Aqua (Life Technologies, Saint-Aubin, France) labeling of dead cells, and the following antibodies: CD3-ECD and CD31-FITC (Beckman Coulter), CD4-APC-efluor780 (e-bioscience, Paris, France), CD45RA-V450 and CD197-PC7 (BD Biosciences, Rungis, France). CD4_RTE_ levels were defined as the percentage of naive CD45RA^+^CCR7^+^CD4^+^ T lymphocytes positive for CD31.

### Virological assays

*Env* and *RT* gene sequences were determined from HIV DNA in the blood, as described elsewhere (http://www.hivfrenchresistance.org/). Phylogenetic analyses were carried out on the sequences of the *Env* and *RT* genes, to determine virus subtype. The phylogenetic relationships between the *RT* sequences were evaluated by comparison with sequences from previously reported representatives of group M, including reference sequences for subtypes and all the CRF sequences available from the HIV database or GenBank (http://www.hiv-web.lanl.gov). Clustal X was used for sequence alignment. Phylogenetic trees were constructed by the neighbor-joining method, and the reliability of the branching order was evaluated with Clustal X, by a bootstrapping approach (1000). Bootstrap values ≥ 70% were considered to provide significant support for membership of a subtype or a CRF. HIV-1 coreceptor usage was determined with the SVMGeno2pheno algorithm, with a 10% false-positive rate [21]. HIV-RNA levels were quantified at clinical sites, whereas HIV-DNA levels were quantified by real-time PCR with the GENERIC HIV-DNA kit (Biocentric, Bandol, France) at a centralized laboratory [22, 23].

### Statistical analysis

We used Fisher’s exact test to compare the HIV characteristics of patients classified on the basis of their Gag-specific CD4 and CD8 T-cell proliferation. Logistic regression was used for univariate and multivariate analyses of data. HIV-RNA levels reflect the degree of antigenic stimulation, which has a major impact on the proliferative capacity of virus-specific CD4 and CD8 T cells [24]. We therefore carried out analyses separately for treated aviremic patients (*n* = 53) and viremic patients (*n* = 26). We took ethnic origin into account, by grouping together patients whose mothers originated from sub-Saharan Africa and the Caribbean and comparing these patients with those whose mothers came from elsewhere (mainland France, North Africa and Asia). Immunological history parameters were defined with CD4 T-cell percentages because these percentages display lower levels of age-related variation than CD4 T-cell counts. Before carrying out multivariate analyses, we checked that there were no interactions between ethnicity and Gag-specific T-cell proliferation for variables with a *P* value <0.20 (data not shown) in univariate analyses: we carried out an ANOVA for continuous variables and we assessed the variation of the OR across the strata for categorical variables. These analyses were exploratory and were not, therefore, corrected for multiple comparisons. For multivariate analyses, sex, ethnicity and HIV subtype were included as exposure factors of interest, regardless of their *P* value. Other variables were included if associated with Gag-specific T-cell proliferation, with a *P* value <0.20 in univariate analysis. Collinear variables not included in the models are listed in the table’s footnote. Analyses were conducted with SAS software (version 9.2). A *P* value of <0.05 was considered to indicate statistical significance.

## Results

### Patients

The ANRS-EP38-IMMIP study included 93 patients who acquired HIV-1 infection during the perinatal period and were at least 15 years old at the time of the study [23]. A single blood sample was drawn for virological and immunological assays. The analyses described here were restricted to the 79 patients for whom a valid proliferation assay had been carried out. Thirty-five of the patients were male. Thirty of the patients were black and 48 came from other ethnic groups. One patient was adopted and of unclear ethnicity. At the time of the study, the patients were 15 to 24 years old (median: 18 years, interquartile range (IQR): 15–19 years). Eleven patients were untreated, whereas 68 were receiving highly active antiretroviral treatment (HAART). Fifty-three patients had undetectable levels of HIV-RNA in plasma. The aviremic patients had been on HAART for a median cumulative duration of 9.1 years (IQR: 7.6–9.8 years) at the time of the study. They had a median CD4 T-cell count of 635 cells/μl (IQR: 521–918 cells/μl). Three of the 26 viremic patients had never received HAART, eight were off HAART (having been treated before) and 15 were on HAART. At the time of the study, the viremic patients had a median CD4 T-cell count of 436 cells/μl (IQR: 316–577 cells/μl) and a median viral load of 3.4 log_10_ HIV RNA copies/ml (IQR: 2.9–3.9 4 log_10_ HIV RNA copies/ml). Fifty-six patients were infected with a virus carrying subtype B sequences for both *Env* and *RT*. Twenty-two patients were infected with viruses carrying a non-B subtype sequence for at least one of these genes, and were considered to be infected with a non-subtype B virus. Information was missing for one patient. Forty-three patients were infected with viruses using CCR5 as a coreceptor, 30 were infected with viruses using CXCR4 as a coreceptor or a dual-tropic virus, and this information was missing for six patients.

### Low frequencies of Gag-specific T-cell proliferation in infected youths

**Fig 1:**
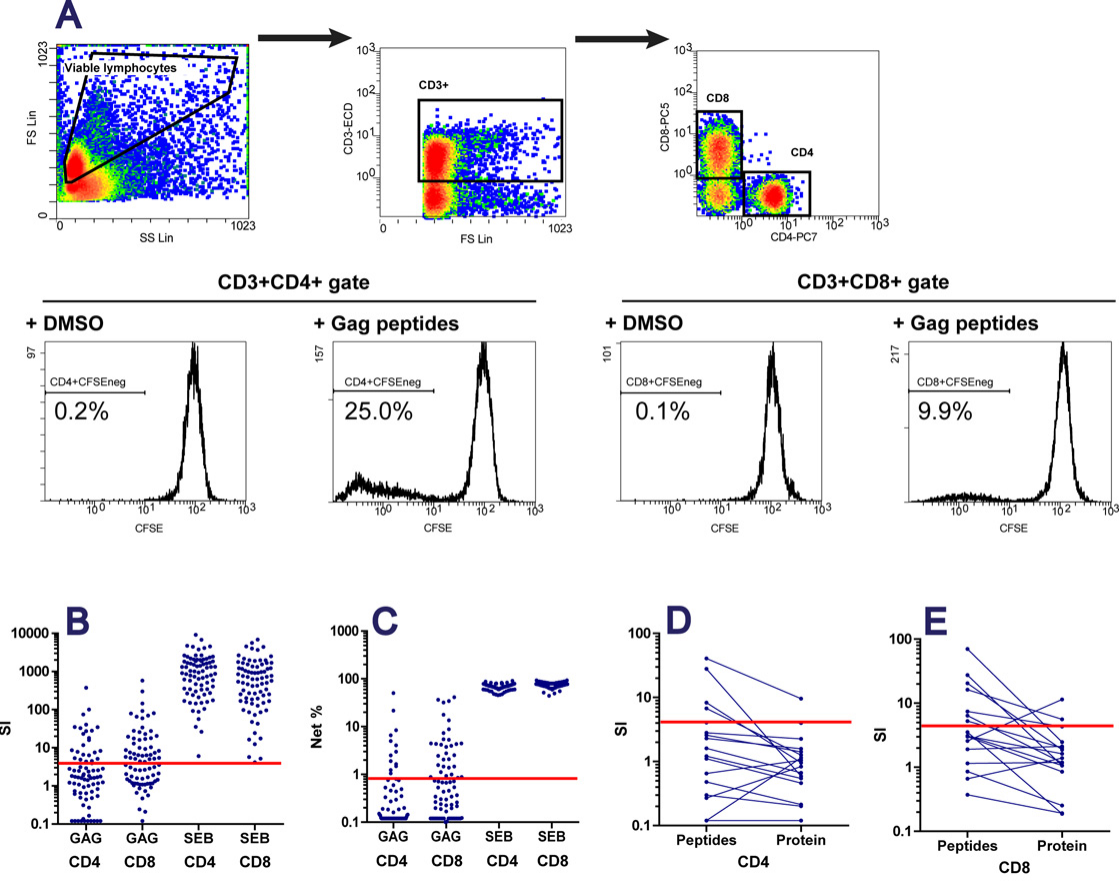
CSFE-based assay for detecting Gag-specific T-cell proliferation. A. The gating strategy involved a FS-SS gate for viable lymphocytes, with the exclusion of FSC^lo^-SSC^int^ apoptotic cells; a FS-CD3 gate for CD3^+^ lymphocytes; CD4^+^CD8^-^ and CD4^-^CD8^+^ gates for CD4 and CD8 T lymphocytes, respectively; a CFSE^low^ gate for proliferating CD4 and CD8 T lymphocytes. For the 79 patients studied, SI (panel B) and net difference in CFSE^low^ percentages (panel C) in response to the Gag peptide pool and SEB, used as positive control, are shown. For 17 patients, the assay was carried out against both the Gag peptide pool and p24^gag^ protein (used as described in [47]); SI values are shown for CD4 and CD8 T-cell responses (panels D and E, respectively). On panels B to E, the red bars indicate the positivity threshold.

We used a CFSE-based assay to quantify T-cell proliferation in response to a pool of Gag peptides corresponding to the clade B consensus sequence (Fig 1A). Taking proliferation in wells stimulated with peptide diluent into account, we calculated Gag-specific proliferation as a stimulation index (SI) and as a difference in the % of CFSE^low^ cells (Fig 1B and 1C). The proliferation of PBMCs from uninfected donors was taken as a reference to define the threshold for positive responses, as described in the materials and methods section. Twelve (16%) of the 79 patients had detectable Gag-specific CD4 T-cell proliferation (CD4 responders, CD4Rs) and 30 (38%) had detectable Gag-specific CD8 T-cell proliferation (CD8 responders, CD8Rs). All patients responded to SEB, used as a positive control (Fig 1B and 1C). Eleven of the 12 CD4Rs were also CD8Rs. Median (interquartile range, IQR) SI values were 30 (9–66) and 20 (7–66) for CD4Rs and CD8Rs, respectively; median (IQR) values for net percentages of CFSE^low^ cells were 3.5 (1.7–7.9) and 3.2 (1.3–7.7) for CD4Rs and CD8Rs, respectively.

We conducted additional experiments to check that the low frequencies of responders were not due to our experimental procedures. Stimulation with the Gag peptide pool resulted in higher levels of T-cell proliferation than stimulation with the recombinant p24^gag^ protein, the most commonly used Gag antigen in T-cell proliferation assays, in a subset of 17 patients tested with both antigens (Fig 1D and 1E). PBMCs were tested in an intracellular cytokine assay, and 62 of 72 patients displayed detectable IFN-γ and/or IL-2 production in response to the Gag peptide pool (data not shown). Over the same period, we consistently detected CD4 T-cell proliferation in response to tetanus toxoid in more than 80% of PBMC samples from uninfected subjects, with a median (IQR) SI of 37(37–116). These data indicate that the low frequency of patients with detectable Gag-specific T-cell proliferation was not related to inadequate antigen or in vitro culture conditions. Instead, it was a characteristic of our study population.

### Being black was strongly associated with Gag-specific T-cell proliferation

We studied factors associated with Gag specific T-cell proliferation. Black patients were more likely to be CD4Rs or CD8Rs than patients from other ethnic groups (CD4: 32% vs. 4%, *P* = 0.001; CD8: 55% vs. 26%, *P* = 0.02, Table 1). Aviremic patients were more likely to be CD4Rs than viremic patients, and male patients were more likely be CD4Rs than female patients, but these differences were not significant (Table 1). Neither viremia at the time of the study nor sex was associated with Gag-specific CD8 T-cell proliferation (Table 1).

**Table 1:** Black ethnicity was strongly associated with Gag-specific CD4 and CD8 T-cell proliferation.

		CD4NRs	CD4Rs		CD8NRs	CD8Rs	
		*n* = 67	*n* = 12		*n* = 49	*n* = 30	
		% (n)	% (n)	*P* ^*a*^	% (n)	% (n)	*P* ^*a*^
Plasma HIV-RNA							
	Undetectable	79.8 (42)	20.8 (11)	0.09	58.5 (31)	41.5 (22)	0.46
	Detectable	96.2 (25)	3.8 (1)		69.2 (18)	30.8 (8)	
Sex							
	Male	77.1 (27)	22.9 (8)	0.12	54.3 (19)	45.7 (16)	0.25
	Female	90.9 (40)	9.1 (4)		68.2 (30)	31.8 (14)	
Ethnicity							
	Black	67.7 (20)	32.3 (10)	0.001	45.2 (14)	54.8 (16)	0.02
	Other	95.7 (45)	4.3 (2)		74.5 (35)	25.5 (12)	
HIV-1 subtype							
	B	87.5 (49)	12.5 (7)	0.30	66.1 (37)	33.9 (19)	0.44
	Non-B	77.3 (17)	22.7 (5)		54.6 (12)	45.4 (10)	

^a^ Fisher’s exact test *P* value.

The perinatally infected patients born in France were infected with viruses of the B subtype and diverse non-B subtypes, reflecting the diverse geographic origins of their mothers [25]. Proliferation assays were carried out on fresh blood samples before characterization of the infecting subtype. Given the high degree of cross-recognition of HIV proteins by HIV-specific T cells from French pediatric patients [25, 26], we used peptides corresponding to the HIV-1 B consensus sequence to stimulate PBMCs from all patients. We were able to sequence the *env* and/or *RT* genes of 78 of the 79 patients, and 56 patients were found to be infected with a subtype B virus. In analyses of the entire group of patients, we found no association between HIV-1 subtype and Gag-specific T-cell proliferation (Table 1). In analyses of the 30 black patients infected with known HIV-1 subtypes, 50% of the patients infected with a subtype B virus and 25% of those infected with a non-B subtype virus were CD4Rs (Fisher’s exact test *P* = 0.23). We found that 60% of the black patients infected with a subtype B virus and 50% of those infected with a non-B subtype virus were CD8Rs (*P* = 0.71). Only two of the 48 patients from other ethnic groups were infected with a non-B subtype virus, precluding analysis. An underestimation of the frequency of CD4Rs among patients infected with non-B subtype viruses in our study is therefore possible, but was not statistically significant.

### In aviremic patients, Gag-specific CD4 T-cell proliferation was associated with ethnicity

We then analyzed the association between Gag-specific CD4 T-cell proliferation and HIV disease history in the 53 aviremic patients, 11 of whom were CD4Rs. We hypothesized that the initiation of effective treatment at younger ages would have a positive impact on the restoration of HIV-specific T-cell levels. We considered two variables, age at first HAART and age at the time of nadir CD4 T-cell percentage, the second of these variables being a proxy for the initiation of effective treatment restoring the immune system. CD4Rs were younger than CD4NRs at the time of nadir CD4 T-cell percentage (median ages: 6 vs. 8 years, respectively, *P* = 0.05, Table 2). In addition, CD4Rs tended to be younger at the time of first HAART than CD4NRs. However, CD4Rs and CD4NRs were of similar age at the time of the study. A younger age at the initiation of treatment-induced CD4 T-cell recovery may be associated with a more robust reconstitution of naive T cells and functional virus-specific T lymphocytes. However, at the time of the study, CD4Rs and CD4NRs had similar levels of naive CD4 T lymphocytes and CD4 recent thymic emigrants (Table 2).

**Table 2:** Analyses of association between HIV disease history and Gag-specific CD4 T-cell proliferation in aviremic patients.

	CD4NRs	CD4Rs	Unadjusted analysis		Adjusted analysis	
	% (n)	% (n)	OR [95% CI]	*P* ^a^	OR [95% CI]	*P* ^b^
	Median (IQR)	Median (IQR)				
**Sociodemographic & current status**						
Sex						
Male	70.8 (17)	29.2 (7)	Reference	0.18	Reference	0.19
Female	86.2 (25)	13.8 (4)	0.39 [0.10–1.54]		0.23 [0.03–2.02]	
Ethnicity						
Black	52.6 (10)	47.4 (9)	Reference	0.002	Reference	0.008
Other	94.1 (32)	5.9 (2)	0.07 [0.01–0.38]		0.02 [0.00–0.37]	
CDC stage						
Non-C	81.6 (31)	18.4 (7)	Reference	0.51		
C	73.3 (11)	26.7 (4)	1.61 [0.39–6.58]			
HIV-1 subtype						
B	85.4 (35)	14.6 (6)	Reference	0.04	Reference	0.55
Non-B	54.6 (6)	45.5 (5)	4.86 [1.12–21.12]		1.93 [0.22–17.13]	
HIV-1 coreceptor usage ^c^						
R5	88.5 (23)	11.5 (3)	Reference	0.10	Reference	0.39
X4R5	68.2 (15)	31.8 (7)	3.58 [0.80–16.05]		2.45 [0.31–19.06]	
Age ^d^	18 (16–20)	17 (15–18)	0.76 [0.55–1.07]	0.11		
HIV-DNA ^e^	2.7 (2.5–3.0)	2.8 (2.1–3.0)	0.82 [0.22–2.99]	0.76		
CD4 T-cell count/μl	635 (546–867)	675 (500–949)	1.00 [0.99–1.00]	0.66		
CD4 T-cell percentage	31 (29–34)	31 (23–35)	0.94 [0.86–1.04]	0.23		
CD8 T-cell count/μl	628 (549–778)	756 (500–944)	1.00 [0.99–1.00]	0.70		
CD8 T-cell percentage	39 (32–44)	36 (32–41)	0.96 [0.88–1.04]	0.33		
CD4/CD8 ratio	0.83 (0.65–1.12)	0.92 (0.57–0.98)	1.26 [0.21–7.16]	0.80		
Naive CD4 T cells ^f^	55 (44–63)	55 (37–68)	1.00 [0.95–1.06]	0.98		
Naive CD8 T cells ^g^	36 (29–49)	38 (22–58)	1.00 [0.96–1.05]	0.78		
CD4 recent thymic emigrants ^h^	78.2 (73.8–80.0)	73.1 (68.8–83.6)	0.96 [0.86–1.08]	0.54		
**HIV disease history**						
Age at first HAART ^d^	7 (6–10)	5 (4–9)	0.81 [0.63–1.04]	0.09		
Cumulative duration of HAART over the last 10 years ^i^	108 (92–118)	110 (83–116)	1.00 [0.97–1.03]	0.96		
Nadir CD4 T-cell percentage	8 (3–14)	4 (1–13)	0.94 [0.84–1.05]	0.27		
Age at nadir CD4 T-cell percentage ^d^	8 (6–11)	6 (3–9)	0.80 [0.64–0.99]	0.05	0.80 [0.59–1.09]	0.15
Duration for which CD4 T-cell percentage < 15 ^i^	20 (2–50)	32 (12–87)	1.01 [0.99–1.03]	0.28		
Duration of last period for which HIV-RNA <500 copies/ml ^i^	38 (16–75)	29 (16–83)	0.99 [0.98–1.02]	0.94		
Cumulative viremia over the last 10 years ^j^	4734 (1812–6292)	3341 (2235–6614)	0.99 [0.99–1.00]	0.96		
Cumulative viremia over the last 5 years ^j^	436 (0–2636)	656 (0–4142)	1.00 [0.99–1.00]	0.86		

^a^ Analysis was performed by logistic regression

^b^ adjusted for variables included in the model. The following variables with *P* values < 0.20 in univariate analysis were not included in the model, because of their associations with other independent variables: age at the time of the study and age at first HAART were associated with age at nadir CD4 T-cell percentage

^c^ Virions using CCR5 as a coreceptor are referred to as R5 virions, whereas those CXCR4 as a coreceptor and dual-tropic viruses are referred to as X4R5 virions

^d^ expressed in years

^e^ expressed in log_10_ copies per 10^6^ PBMCs

^f^ expressed as a percentage of total CD4 T cells

^g^ expressed as a percentage of total CD8 T cells

^h^ expressed as a percentage of naive CD4 T cells

^i^ expressed in months

^j^ expressed in days x log_10_ HIV-RNA copies/ml of plasma.

We then hypothesized that the intensity (i.e. nadir CD4 T-cell percentage) and duration of the immunodeficiency would be inversely related to the restoration of HIV-specific T-cell levels after the treatment-induced suppression of viral replication. However, CD4Rs and CD4NRs did not differ in terms of nadir CD4 T-cell percentages or cumulative time with a CD4 T-cell percentage < 15% (4% vs. 8%, *P* = 0.27; and 32 vs. 20 months, *P* = 0.28, respectively, Table 2). The degree of immunological recovery at the time of the analysis was assessed by determining absolute CD4 and CD8 T-cell counts, percentages and their ratio. None of these measures was associated with Gag-specific T-cell proliferation (Table 2). Two other indicators of the severity of past HIV disease—a previous CDC stage C event and the presence of X4R5 viruses in PBMCs—were not associated with Gag-specific T-cell proliferation (Table 2).

Finally, we tested the hypothesis that recent re-exposure to viral replication was associated with enhanced Gag-specific T-cell proliferation in patients displaying a suppression of viral replication at the time of the study. We observed that CD4Rs had similar duration of HIV-RNA levels continuously below 500 copies/ml before the time of the analysis than CD4NRs (29 vs. 38 months, *P* = 0.94) and similar HIV DNA levels (2.7 vs. 2.8 log_10_ HIV DNA copies/ml, *P* = 0.76).

In multivariate analysis, Gag-specific CD4 T-cell proliferation was associated with black ethnicity independently of sex, HIV-1 subtype, HIV-1 coreceptor usage and age at nadir CD4 T-cell percentage (Table 2).

### In aviremic patients, Gag-specific CD8 T-cell proliferation was associated ethnicity and shorter duration of viral suppression.

We then analyzed the association between Gag-specific CD8 T-cell proliferation and HIV disease history in the 53 aviremic patients, 22 of whom were CD8Rs. Median age at nadir CD4 T-cell percentage was six years for CD8Rs and eight years for CD8NRs (*P* = 0.04, Table 3). In addition, CD8Rs tended to be younger than CD8NRs at the time of the study, but no difference was observed between these two groups of patients in terms of naive CD4 T-cell, CD4 recent thymic emigrants, and naive CD8 T-cell percentages (Table 3). CD8Rs and CD8NRs had similar nadir CD4 T-cell percentages and durations of severe immunosuppression (4% vs. 10%, *P* = 0.16; and 35 vs. 14 months, *P* = 0.16, respectively). Gag-specific CD8 T-cell proliferation was not associated with current CD4 and CD8 T-cell levels, nor with previous CDC stage C event and the presence of X4R5 viruses in PBMCs (Table 3).

**Table 3:** Analysis of association between HIV disease history and Gag-specific CD8 T-cell proliferation in aviremic patients.

	CD8NRs	CD8Rs	Unadjusted analysis		Adjusted analysis	
	% (n)	% (n)	OR [95% CI]	*P* ^a^	OR [95% CI]	*P* ^b^
	Median (IQR)	Median (IQR)				
**Sociodemographic & current status**						
Sex						
Male	54.2 (13)	45.8 (11)	Reference	0.56	Reference	0.99
Female	62.1 (18)	37.9 (11)	0.72 [0.24–2.17]		0.99 [0.25–3.97]	
Ethnicity						
Black	32.2 (6)	68.4 (13)	Reference	0.004	Reference	0.05
Other	73.5 (25)	26.5 (9)	0.17 [0.05–0.57]		0.18 [0.03–1.00]	
CDC stage						
Non-C	63.2 (24)	36.8 (14)	Reference	0.28		
C	46.7 (7)	53.3 (8)	1.96 [0.58–6.57]			
HIV-1 subtype						
B	65.9 (27)	34.1 (14)	Reference	0.09	Reference	0.87
Non-B	36.3 (4)	63.7 (7)	3.38 [0.84–13.52]		0.86 [0.13–5.79]	
HIV-1 coreceptor usage ^c^						
R5	57.7 (15)	42.3 (11)	Reference	0.92		
X4R5	59.1 (13)	40.9 (9)	0.94 [0.30–2.99]			
Age ^d^	18 (16–21)	17 (15–18)	0.80 [0.62–1.03]	0.09		
HIV-DNA ^e^	2.8 (2.5–3.0)	2.7 (2.0–3.0)	0.50 [0.16–1.52]	0.22		
CD4 T-cell count/μl	676 (588–929)	592 (494–918)	0.99 [0.99–1.00]	0.67		
CD4 T-cell percentage	31 (30–36)	31 (23–34)	0.95 [0.86–1.02]	0.17		
CD8 T-cell count/μl	644 (513–793)	718 (524–854)	1.00 [0.99–1.00]	0.74		
CD8 T-cell percentage	39 (33–41)	38 (32–44)	0.99 [0.93–1.06]	0.81		
CD4/CD8 ratio	0.83 (0.71–1.03)	0.79 (0.61–1.05)	0.73 [0.15–3.51]	0.70		
Naive CD4 T-cell % ^f^	56.0 (44.5–64.7)	53.7 (40.4–62.1)	0.97 [0.93–1.02]	0.28		
Naive CD8 T-cell % ^g^	42.7 (30.4–52.6)	35.6 (24.4–44.9)	0.98 [0.94–1.02]	0.26		
CD4 recent thymic emigrant % ^h^	76.8 (73.8–79.0)	77.1 (70.0–83.2)	1.00 [0.91–1.10]	0.99		
**HIV disease history**						
Age at first HAART ^d^	8 (7–11)	6 (5–9)	0.90 [0.76–1.06]	0.20		
Cumulative duration of HAART over the last 10 years ^i^	109 (87–118)	108 (98–116)	1.00 [0.98–1.03]	0.75		
Nadir CD4 T-cell percentage	10 (4–14)	4 (2–13)	0.94 [0.86–1.03]	0.16		
Age at nadir CD4 T-cell percentage ^d^	8 (7–10)	6 (4–10)	0.84 [0.71–0.99]	0.04	0.84 [0.69–1.02]	0.09
Duration for which CD4 T-cell percentage < 15 ^i^	14 (2–45)	35 (12–62)	1.01 [0.99–1.03]	0.16		
Duration of last period for which HIV-RNA <500 copies/ml ^i^	54 (22–83)	20 (8–57)	0.98 [0.97–0.99]	0.04	0.98 [0.96–0.99]	0.05
Cumulative viremia over the last 10 years ^j^	4506 (1459–6292)	4295 (2235–6615)	1.00 [0.99–1.00]	0.79		
Cumulative viremia over the last 5 years ^j^	317 (0–2636)	781 (1664–2934)	1.00 [0.99–1.00]	0.77		

^a^ Analysis was performed by logistic regression

^b^ adjusted for variables included in the model. The following variables with *P* values < 0.20 in univariate analysis were not included in the model, because of their associations with other independent variables: age at the time of the study was associated with age at nadir CD4 T-cell percentage; CD4 T-cell percentage, nadir CD4 T-cell percentage and duration for which CD4 T-cell percentage <15 were associated with ethnicity

^c^ Virions using CCR5 as a coreceptor are referred to as R5 virions, whereas those CXCR4 as a coreceptor and dual-tropic viruses are referred to as X4R5 virions

^d^ expressed in years

^e^ expressed in log_10_ copies per 10^6^ PBMCs

^f^ expressed as a percentage of total CD4 T cells

^g^ expressed as a percentage of total CD8 T cells

^h^ expressed as a percentage of naive CD4 T cells

^i^ expressed in months

^j^ expressed in days x log_10_ HIV-RNA copies/ml of plasma.

In contrast to CD4 responses, we observed that CD8Rs had a significantly shorter duration of HIV-RNA levels continuously below 500 copies/ml before the time of the analysis than CD8NRs (20 vs. 54 months, *P* = 0.04). However, cumulative viremia over the last 5 and 10 years before the study and cell-associated HIV DNA levels did not differ between CD8Rs and CD8NRs (Table 3).

In multivariate analysis, Gag-specific CD8 T-cell proliferation was independently associated with black ethnicity and shorter duration of the last period of HIV RNA levels < 500 copies/ml after adjustment for sex and HIV-1 subtype (Table 3). There was also a trend for association with a younger age at nadir CD4 T-cell percentage. In univariate analysis, current and nadir CD4 T-cell percentages, and the duration of severe immunosuppression tended to be weakly associated with Gag-specific CD8 T-cell proliferation. These CD4 T cell-related variables were associated with ethnicity and were excluded from the initial model, to prevent overadjustment. In sensitivity analyses, we found that the inclusion of any of these three CD4 T cell-related variables had only a modest impact on the association between Gag-specific CD8 T-cell proliferation, ethnicity, duration of viral suppression and age at nadir CD4 T-cell percentage (data not shown).

### In viremic patients, Gag-specific CD8 T-cell proliferation was associated with lower levels of HIV replication

One of the 26 patients with detectable levels of HIV-RNA at the time of the study was a CD4R and eight were CD8Rs. We therefore focused our analysis on CD8 T-cell proliferation. Eleven patients were untreated and 15 patients were on HAART at the time of the study. Gag-specific CD8 T-cell proliferation was associated with lower levels of HIV replication in viremic patients. CD8Rs had significantly lower plasma HIV-RNA levels than CD8NRs (2.7 vs. 3.7 log_10_ HIV-RNA copies/ml, *P* = 0.02, Table 4), and tended to have lower HIV-DNA levels in their PBMCs (2.8 vs. 3.2 log_10_ HIV-DNA copies/10^6^ PBMCs, *P* = 0.08). Furthermore, HIV RNA levels before the study were lower in CD8Rs than in CD8NRs, with significant differences observed for cumulative viremia over the last five years (5411 vs. 1248 days x log_10_ HIV-RNA copies/ml, *P* = 0.006).

**Table 4:** Analyses of association between HIV disease history and Gag-specific CD8 T-cell proliferation in viremic patients.

	CD8NRs	CD8Rs	Unadjusted analysis		Adjusted analysis	
	% (n)	% (n)	OR [91% CI]	*P* ^a^	OR [95% CI]	*P* ^b^
	Median (IQR)	Median (IQR)				
**Sociodemographic & current status**						
Sex						
Male	54.5 (6)	45.5 (5)	Reference	0.17	Reference	0.79
Female	80.0 (12)	20.0 (3)	0.30 [0.05–1.70]		1.72 [0.03–104.44]	
Ethnicity						
Black	66.7 (8)	33.3 (4)	Reference	0.57	Reference	0.50
Other	76.9 (10)	23.1 (3)	0.60 [0.10–3.49]		7.59 [0.02–2379]	
CDC stage (non-C vs. C)						
Non-C	70.0 (14)	30.0 (6)	Reference	0.88		
C	66.7 (4)	33.3 (2)	1.17 [0.17–8.19]			
HIV-1 subtype						
B	66.7(10)	33.3(5)	Reference	0.74	Reference	0.66
Non-B	72.7(8)	27.3(3)	0.75 [0.14–4.13]		4.22 [0.00–1911]	
HIV-1 coreceptor usage ^c^						
R5	64.7 (11)	35.3 (6)	Reference	0.26		
X4R5	87.5 (7)	12.5 (1)	0.26 [0.03–2.67]			
Current HAART						
No	81.8 (9)	18.2 (2)	Reference	0.24		
Yes	60.0 (9)	40.0 (6)	3.00[0.47–19.04]			
Age ^d^	17 (15–18)	18 (16–20)	1.20 [0.84–1.73]	0.32		
HIV-RNA ^e^	3.7 (3.2–4.3)	2.7 (2.2–3.0)	0.03 [0.00–0.54]	0.02	0.04 [0.00–0.76]	0.03
HIV-DNA ^f^	3.2 (2.8–3.5)	2.8 (2.6–3.0)	0.14 [0.02–1.29]	0.08		
CD4 T-cell count/μl	436 (313–534)	464 (298–602)	0.99 [0.99–1.00]	0.76		
CD4 T-cell percentage	25 (17–27)	23 (18–28)	0.98 [0.89–1.08]	0.71		
CD8 T-cell count/μl	895 (636–1189)	742 (598–1035)	0.99 [0.99–1.00]	0.42		
CD8 T-cell percentage	54 (47–59)	45 (41–54)	0.94 [0.87–1.03]	0.19		
CD4/CD8 ratio	0.44 (0.31–0.57)	0.48 (0.39–0.72)	0.87 [0.10–7.76]	0.91		
Naive CD4 T-cell % ^g^	62 (56–67)	60 (50–64)	0.95 [0.86–1.04]	0.28		
Naive CD8 T-cell % ^h^	25 (18–30)	22 (18–27)	0.98 [0.88–1.09]	0.71		
CD4 recent thymic emigrant % ^i^	81.0 (78.1–84.4)	83.4 (82.5–90.2)	1.10 [0.92–1.31]	0.30		
**HIV disease history**						
Age at first HAART ^d^	7 (5–9)	9 (6–13)	1.42 [0.97–2.07]	0.07	2.05 [0.77–5.47]	0.15
Cumulative duration of HAART over the last 10 years ^j^	86 (52–117)	77 (69–101)	0.99 [0.98–1.02]	0.85		
Nadir CD4 T-cell %	10 (6–17)	10 (3–12)	0.95 [0.83–1.08]	0.42		
Age at nadir CD4 T-cell % ^d^	7 (3–11)	7 (5–10)	1.00 [0.86–1.17]	0.96		
Duration for which CD4 T-cell % < 15 ^j^	18 (0–45)	34 (8–87)	1.23 [0.99–1.04]	0.16		
Cumulative viremia over the last 10 years ^k^	10779 (6139–11959)	5915 (4654–8020)	0.99 [0.99–1.00]	0.08		
Cumulative viremia over the last 5 years ^k^	5411 (3607–6139)	1248 (492–2823)	0.99 [0.99–0.99]	0.01		

^a^ Analysis was performed by logistic regression

^b^ adjusted for variables included in the model. The following variables with *P* values < 0.20 in univariate analysis were not included in the model, because of their associations with other independent variables: HIV DNA levels, cumulative viremia over the last 10 and 5 years were associated with current HIV RNA levels; duration for which CD4 T-cell % <15 were associated with age at first HAART

^c^ Virions using CCR5 as a coreceptor are referred to as R5 virions, whereas those CXCR4 as a coreceptor and dual-tropic viruses are referred to as X4R5 virions

^d^ expressed in years

^e^ expressed in log_10_ copies/ml of plasma

^f^ expressed in log_10_ copies per 10^6^ PBMCs

^g^ expressed as a percentage of total CD4 T cells

^h^ expressed as a percentage of total CD8 T cells

^i^ expressed as a percentage of naive CD4 T cells

^j^ expressed in months

^k^ expressed as days x log_10_ HIV-RNA copies/ml of plasma.

CD8Rs tended to be older than CD8NRs at the time of HAART initiation (9 vs. 7 years, *P* = 0.07). Age at the time of the study, current CD4 T-cell levels, past immunosuppression and naive T-cell percentages did not differ significantly between CD8Rs and CD8NRs (Table 4). No association was found between treatment status, ethnicity, sex, previous CDC stage C events, virus coreceptor usage and Gag-specific CD8 T-cell proliferation (Table 4).

In multivariate analysis, Gag-specific CD8 T-cell proliferation was significantly associated with current HIV-RNA levels, after adjustment for sex, ethnicity, HIV subtype and age at first HAART (Table 4). In conclusion, Gag-specific CD8 T-cell proliferation was associated with lower levels of HIV replication in patients displaying active viral replication.

## Discussion

This study focused on the first generation of youths with perinatally acquired HIV-1 infection. Their immune system was exposed to persistent viral replication during the first few years of their life, followed by periods of viral suppression of variable duration. The data presented here are the first to be published concerning the long-term outcome of HIV-specific T cells in these patients. Our major findings were as follows: (1) Gag-specific CD4 and CD8 T-cell proliferation was more frequent in black patients than in patients from other groups; (2) Gag-specific CD8 T-cell proliferation was associated with a shorter duration of viral suppression in aviremic patients, and with lower plasma viral loads in viremic patients.

The strong association between ethnicity and HIV-specific T-cell proliferation observed here is the principal and most original finding of this study. Ethnicity was related to T-cell proliferation only in aviremic patients. A few other studies of individuals from various ethnic groups living in a similar environment have reported higher levels of pathogen-specific T-cell responses in black individuals than in individuals from other groups. In vertically infected children living in the US, the frequency of HIV-specific IFN-γ-producing CD8 T cells was found to be higher in African-Americans than in Hispanics [27]. T-cell proliferation in response to HCV was more frequent and targeted more viral proteins in adult African-Americans than in Caucasians [28], and HCV-specific T cells from Caucasians expressed higher levels of the inhibitory molecule PD-1 [29]. A study on HIV-infected patients on suppressive HAART conducted in the United States reported that black patients had stronger proliferative T-cell responses to fungi than white individuals [30]. Among patients with pulmonary tuberculosis followed up in the United Kingdom, those of African ancestry produced larger amounts of inflammatory mediators in response to *Mycobacterium tuberculosis* antigens than those of Eurasian ancestry [31]. However, T-cell responses to mitogens and/or vaccine antigens in black subjects were similar to or weaker than those in individuals from other ethnic groups [28, 32, 33]. Overall, our results and those of several other studies suggest that T-cell responses to persistent infectious agents may be more frequent and/or of higher intensity in black patients than in patients from other ethnic groups.

The higher frequency with which Gag-specific T-cell proliferation was detected in black patients was not due to major differences in HIV infection history. All the patients included in this study had a similar route of infection and duration of HIV disease and all benefited from universal access to care and treatment. Importantly, all but one of these patients were born in France and all had received regular care since birth or for at least 10 years before the start of the study. In the French perinatal cohort, early death, before the age of two years, was more frequent among children born to mothers of African origin than among those born to mothers of other geographic origins [1]. This may have introduced a survivor bias into the analysis presented here. However, the patients who survived until the time of this study were all typical progressors: 60% had reached CDC stages B or C, 80% had had a CD4 T-cell percentage < 15% at least once during follow-up, and 96% had received HAART during their lifetime [23]. Ethnicity was not associated with CDC stage C clinical events, or with receiving HAART (data not shown). Black patients had lower CD4 T-cell counts and percentages at the time of the study and had spent more time with severe immunosuppression than patients from other ethnic groups (data not shown). As lower levels of CD4 T-cells would be expected to be associated with lower levels of Gag-specific T-cell proliferation, the observed association between Gag-specific T-cell proliferation and black ethnicity would have been strengthened by a CD4 T-cell related bias, if such a bias existed.

Ethnicity is related to socioeconomic parameters that may be associated with differences in access to care, even when such care is provided free of charge. Among the youths included in our study, ethnicity was not associated with successful viral suppression or treatment interruption. Among the aviremic patients, the duration of viral suppression was shorter in CD8Rs than in CD8NRs, and such differences were similar in both ethnic groups (i.e. 21 vs. 72 months in patients of black ethnicity and 19 vs. 51 months in patients of other ethnicities). Overall, if socioeconomic factors had any impact on T-cell responses in patients of black ethnicity, this effect was not related to recent virological and treatment history.

Geographic origin is linked to viral subtype. The sequence of the Gag antigen in patients born to mothers from sub-Saharan Africa was different from that of the Gag antigen used in the *in vitro* assay, as there were too many non-B strains circulating in France to test the response to each of them. The rate of detection of T-cell proliferation was lower in patients infected with non-B strains, but the difference was not significant. These results are consistent with the frequent cross-recognition of HIV proteins from different viral subtypes by HIV-specific CD8 T cells from French pediatric patients [25, 26], and with the results of a large study on 250 individuals from four continents that estimated the ratio of Gag-specific T-cell responses to heterologous clades versus homologous clades to be about 0.7 [34]. T lymphocytes from most HIV-infected patients target multiple epitopes on the Gag protein, and we expressed the results qualitatively, to reduce the impact of undetected epitopes. Underestimation of the HIV-specific T-cell response in patients infected with non-B subtype viruses would have decreased the likelihood of detecting a T-cell response in black patients, decreasing the difference observed between ethnic groups.

Gag-specific CD4 T-cell proliferation was detected in 16% of patients, and CD8 T-cell proliferation was detected in 38%. These rates of response to Gag are lower than those reported in several published studies. Two studies reported frequencies of HIV-specific CD4 T-cell proliferation of about 30% in treated children [35, 36]. However, most studies in child and adult patients have reported HIV-specific proliferation in more than half the patients [37–48]. We carefully assessed the experimental parameters potentially leading to lower response rates, as described in the results section. Comparisons across published studies may be influenced by the techniques used. We used a cytometry-based cell-dye dilution method, with independent assessments of proliferation for CD4 and CD8 T cells, whereas most previous studies were based on the incorporation of radioactive thymidine, a technique that cannot differentiate between different phenotypes of proliferating cells. It thus yields aggregate CD4 and CD8 T-cell response rates, which may be higher than those for a single T-cell subset. The patients studied here had a longer duration of infection and/or exposure to suppressive HAART than the patients described in most published studies, and this provides a probable explanation for the modest frequency of patients with detectable Gag-specific T-cell proliferation.

Exposure to HIV replication was the only HIV disease history-related factor associated with Gag-specific CD8 T-cell proliferation. We found that aviremic CD8Rs did, indeed, have a shorter duration of HIV RNA suppression than aviremic CD8NRs. An increase in HIV-specific T-cell responses following autovaccination due to the production of viral antigens during treatment interruptions has consistently been reported in many studies in adults and children [11, 36, 49–56]. However, no benefit in terms of further virological control or disease progression could be clearly demonstrated [49–51]. In viremic patients, current and past viral loads were lower in CD8Rs than in CD8NRs. This observation is consistent with previous findings for younger HIV-infected children, in which we quantified the IFN-γ production or cytolytic activities of CD8 T cells [57, 58]. Overall, our results suggest that the relationship between exposure to viral replication and the magnitude of HIV-specific T-cell responses yields a bell-shaped curve. The prolonged suppression of viral replication leads to a contraction of the HIV-specific T-cell compartment, whereas viral replication reduces the size of the HIV-specific T-cell compartment by direct killing, indirect effects mediated by immunosuppressive viral proteins, and by inducing the differentiation of these cells into effector cells with antiviral activity but an inability to proliferate.

Our data raise questions about the contribution of T-cell proliferative responses to the maintenance of the viral reservoir. Stronger proliferative responses may help to control viral replication. However, there was a trend toward lower levels of cell-associated HIV-DNA only in CD8Rs from the viremic group. As discussed above and in [59], viral replication is both an inducer and a target of antiviral T cells, and associations between these parameters are difficult to interpret. Studies of the capacity of proliferating HIV-specific T cells to inhibit autologous viral replication may be of interest in these youths. Conversely, HIV-specific CD4 T cells are the preferential targets of HIV viruses and their stimulation may fuel the viral reservoir [60]. The increase in levels of monotypic HIV sequences during long-term suppressive cART in infected children is consistent with the proliferation of infected cells contributing to HIV persistence in these patients [61]. In our study, HIV DNA levels were not significantly associated with the Gag-specific CD4 T-cell response (Table 2) or with ethnicity [23]. Black patients more frequently displayed Gag-specific T-cell proliferation, and TT-specific T-cell proliferation, but their responses to SEB were similar to those of patients from other ethnic groups (data not shown). It remains unclear whether the antigen-specific T-cell responses of blood cells quantified *in vitro* reflect *in vivo* proliferation in all lymphoid compartments. The distinctive features of HIV-specific T-cell responses in youths living with HIV since the perinatal period highlight the need for further studies.

This study has several strengths and limitations. It is one of the first studies to characterize CD8 T-cell proliferation, as previous investigations have focused on the assessment of other CD8 T-cell functions, or did not distinguish between CD4 and CD8 T-cell responses. It is limited by the small number of patients with detectable T-cell proliferation included and its modest statistical power. Our main finding is an association between Gag-specific proliferation and ethnicity. This demographic variable (ethnicity) is related to several factors affecting HIV disease, including viral, socioeconomic and cultural factors, and the genetics and physiology of the host. We determined viral subtype for the patients studied here. HIV disease parameters potentially affected by sociodemographic factors were included in the statistical analyses and are discussed. Unfortunately, there were no genetic studies, and no HLA-tissue typing, as specific consent is required for genetic investigations in France. We did not include such tests as a requirement for inclusion in the study, as we were concerned that this would have reduced the willingness of subjects to participate. Ethnicity was defined by the geographic origin of the mother only, which was known for all but one patient, who was adopted. The geographic origin of the father was not known for all patients. Mixed ethnic origin was rare in the population studied, and the definition of ethnicity on the basis of the mother’s geographic origin alone was therefore considered appropriate. Another limitation was that the viremic group comprised patients with three treatment statuses (HAART naive, HAART interruption and on HAART), because the small numbers of patients in the three separate groups precluded independent analysis. Active viral replication at the time of the study led to differences in the relationships between HIV-specific T-cell responses and HIV disease history. We therefore felt that it was important to present the data for the viremic and aviremic groups. However, the results for the viremic group should be interpreted with caution due to the heterogeneous nature of this group.

Our results reveal differences in T-cell responses related to ethnicity, for youths born in the same country, with similar access to care. This result is important, as most infected children live in sub-Saharan Africa, where the number of adolescents with long-term exposure to HIV replication is growing [62]. HIV pathogenesis studies are used as a basis for designing immune-based interventions, and our results clearly highlight the need to take ethnicity into account in such studies. The impact of HIV virological history on Gag-specific T-cell proliferation in our patients appeared to be similar to that reported for adults. However, the lack of association between T-cell function and CD4 T-cell history is a key difference between this population and patients infected as adults. This difference was particularly striking for black youths, who displayed more frequent T-cell proliferative responses despite having had more severe immunosuppression in the past. From a clinical perspective, T lymphocytes specific for pathogens and tumor antigens would be likely to influence the development of morbidity and mortality in these patients. It would be of interest to study their functional restoration in these patients, together with the associated demographic and clinical correlates, and to design appropriate vaccine-based prevention approaches. We now aim to carry out immunological studies to identify the mechanisms underlying the more vigorous HIV-specific T-cell proliferation in black patients.

## Appendix

The institutions and investigators of the ANRS-EP38-IMMIP Study were: Pédiatrie-néonatologie, Hôpital Louis Mourier, Colombes (Corinne Floch-Tudal); Gynécologie-Obstétrique, Groupe Hospitalier Cochin Tarnier-Port-Royal, Paris (Ghislaine Firtion); Pédiatrie-Centre Hospitalier Intercommunal, Créteil (Sophie Lemerle); Pédiatrie-Centre Hospitalier Intercommunal, Villeneuve Saint-Georges (Anne Chace); Immuno-Hématologie Pédiatrique, Groupe Hospitalier Necker-Enfants Malades, Paris (Stéphane Blanche, Florence Veber); Pédiatrie, Centre Hospitalier Sud-Francilien, Evry (Adrien May); Maladies Infectieuses, Hôpital Jean Verdier, Bondy (Vincent Jeantils); Onco-Hématologie Pédiatrique Hôpital Trousseau, Paris (Catherine Dollfus); Pédiatrie-Hôpital Robert Debré, Paris (Martine Levine, Albert Faye); Centre de Diagnostic et de Thérapeutique, Hôpital de l’Hôtel-Dieu, Paris (Jean-Paul Viard).
